# The association between dietary intake and cardiometabolic risk factors among obese adolescents in Indonesia

**DOI:** 10.1186/s12887-022-03341-y

**Published:** 2022-05-12

**Authors:** Indah K. Murni, Dian C. Sulistyoningrum, Rina Susilowati, Madarina Julia, Kacie M. Dickinson

**Affiliations:** 1 Department of Child Health, DR. Sardjito Hospital/Faculty of Medicine, Public Health and Nursing, Universitas Gadjah Mada, Yogyakarta, 55284 Indonesia; 2 Department of Nutrition and Health, Faculty of Medicine, Public Health and Nursing, Universitas Gadjah Mada, Yogyakarta, Indonesia; 3 Department of Histology and Cell Biology, Faculty of Medicine, Public Health and Nursing, Universitas Gadjah Mada, Yogyakarta, Indonesia; 4 Caring Futures Institute, College of Nursing and Health Sciences, Flinders University, Adelaide, Australia

**Keywords:** Dietary intake, Cardiovascular disease, Obese, Adolescents, Indonesia

## Abstract

**Background and objective:**

Poor diets, characterized by excess fat, sugar and sodium intakes, are considered to be one of the most important modifiable risk factors for cardiovascular disease. Diet patterns and intakes during adolescence may persist into adulthood and impact on risk for chronic disease later in life. We aimed to evaluate the dietary intake of obese adolescents and its relationship to cardiometabolic health including lipid status and glycemic control.

**Methods and study design:**

This was a cross-sectional study of obese children aged 15 to < 18 years in Yogyakarta, Indonesia. All children had a medical history performed including a physical examination and fasting blood sample. Dietary intake was assessed using a semi-quantitative recall food frequency questionnaire. Multivariable linear regression model was performed to determine the relationship between dietary intakes and cardiovascular disease risks and to adjust for potential confounders.

**Results:**

Of 179 adolescents, 101 (57.4%) were male and median age was 16.4 (15.0–17.9) years. The majority of adolescents (98%) had inadequate intake of fibre and exceeded intakes of total fat (65%) and total sugar (36%). There was statistically significant correlation found in the multivariable linear regression analysis between fibre intake and HDL cholesterol after adjusting for potential confounders (β = 0.165; *p* = 0.033).

**Conclusions:**

This study demonstrates that there is a high proportion of obese Indonesian adolescents with poor dietary intakes. There was relationship observed between intake of nutrients of concern (fibre) and cardiometabolic risk factor among this sample of obese adolescents. Future research should examine overall dietary patterns in more detail among this population to elucidate the role of poor diet intakes in development of cardiovascular disease risk factors in young people transitioning into adulthood.

**Supplementary Information:**

The online version contains supplementary material available at 10.1186/s12887-022-03341-y.

## Introduction

Obesity is a significant public health priority worldwide [1]. The prevalence of obesity is high and is reported to have increased in low- and middle-income countries including in Indonesia [2, 3]. After 5 years, the prevalence of obesity increased from 1.9% in childhood to 3.2% in adolescence in a Yogyakarta city, Indonesia [2]. More than a decade in the same city in Indonesia, the prevalence of obesity among adolescents is doubling accounting for 7% [3]. The majority of obesity in adulthood originates in childhood and tracks into morbidity later in life [4]. It contributes to the development of risk factors for early cardiovascular diseases including hypertension, metabolic diseases, and atherosclerosis [5]. Identification of factors related to poor cardiometabolic health has been emphasized in children particularly in preventing modifiable risk factors.

It is considered that eating habits established in childhood and adolescence can persist into adulthood [6, 7]. A varied and balanced diet and the establishment of healthy eating habits will promote the health of young people throughout their lives [8]. Poor dietary intake, which is now considered as one of the most important modifiable risk factors for cardiovascular disease, is associated with the development of cardiovascular disease worldwide [8]. Therefore, early modification of eating habits, healthy dietary intake, and body weight can promote health and reduce the risk of developing cardiovascular diseases later in life.

Studies on the association between dietary intake and cardiometabolic risk factors in children are limited. A previous systematic review of 11 studies conducted among Korean samples revealed that there were significant associations between obesity and dyslipidemia with excess intake of nutrients such as sodium, fat, and sugar. All studies focused on adults, but one was undertaken on children aged 9 to 10 years [9]. Another systematic review in adults evaluated the association between coronary heart disease and dietary factors including intake of vegetables, nuts, monounsaturated fatty acids, foods with a high glycemic index, trans–fatty acids, and overall diet quality and dietary patterns. This concluded that only a Mediterranean dietary pattern was causally protective against coronary heart disease [10].

Previous studies on associations between obesity and other cardiovascular disease risks and intake of fat and sugar and dietary fibre intake are conducted in high-income countries [9, 11, 12]. A recent published study evaluated fast food and soda pattern associated with significantly elevated insulin resistance among children and adolescents [11]. Children and adolescents in the high intake of fast food and soda pattern were associated with a higher waist circumference (β = 1.55), insulin level (β = 1.25), and body mass index (β = 0.53) and this was positively associated with HOMA-IR ≥ 2.6 (OR = 2.11; 95% CI: 1.227–3.638) (*p* < 0.05) compared with those in the lower intake of fast food and soda pattern [11]. A previous cross-sectional study was conducted in children evaluating the association between eating patterns and overweight status in children who participated in the Bogalusa Heart Study and found that several eating patterns including sweetened beverages, sweets, meats, and total consumption of low-quality foods were associated with overweight status with OR (95% CI) 1.33 (1.12–1.57), 1.21 (1.00–1.46), 1.38 (1.12–1.71) and 1.35 (1.08–1.68), respectively [12].

There is limited data from low-to-middle income countries, which are experiencing a nutrition transition and increasing prevalence of cardiovascular disease risks in children [13, 14]. Therefore, studies are needed to better identify the relationship between dietary intake and cardiovascular disease risks among obese adolescents. This study aimed to explore the relationship between excess intakes of nutrients of concern and cardiovascular disease risk factors among urban-dwelling adolescents in Indonesia. This is of importance to formulate an effective prevention strategy on dietary intake management for the development of cardiovascular diseases in obese adolescents in Indonesia as a model in low-to-middle income countries. We aimed to evaluate the dietary intake (sugar, fat, and fibre) of obese adolescents and its relationship to cardiovascular disease risk factors particularly impaired lipid profile (HDL and triglyceride) and glycemic control (HbA1c and fasting plasma glucose).

## Materials and methods

### Study design and population

We conducted a cross-sectional study of adolescents in Yogyakarta-Indonesia from February to October 2016. Screening for obesity was performed in seven public and three private high schools in the city [3]. Inclusion criteria were obese adolescents aged 15 to less than 18 years. Exclusion criteria was diagnosis of type 2 diabetes mellitus, renal diseases, cardiac diseases, any history of systemic disease or acute infections or history of current steroid use. Approval for the study was obtained from the Ethics committee of the Faculty of Medicine, Universitas Gadjah Mada, Yogyakarta, Indonesia (KE/FK/0481/EC). Written informed consent was obtained from all parents/guardians of the children included in the study.

### Data collection

All eligible children underwent an interview about their general health and medical history and physical examination and a fasting blood collection. We collected demographic data, histories of tobacco smoke exposure and daily physical activity using a structured questionnaire. Semi quantitative dietary recall was used to estimate the usual diet intake including the intake of carbohydrate, sugar, fibre, protein and fat [15]. The physical activity was measured using international physical activity questionnaire (IPAQ) (http://sites.google.com/site/theipaq/).

We performed a clinical examination, including body weight, height, and waist circumference. We measured the child’s weight using a portable weighing scale (CAMRY, EB9003). All children were weighed with light clothing without shoes or slippers. The weight was recorded as kilograms (kg) to the nearest 0.1 kg. We measured the height using a portable stadiometer or microtoise (GEA) with an erect position. The height was recorded in centimetres (cm) to the nearest of 0.5 cm. BMI was calculated based on weight in kg divided by height in metres squared. As previously described [14], to be considered obese, the adolescents must meet all three obesity criteria of the World Health Organization (WHO) [16], the Centers for Disease Control and Prevention (CDC) [17], and the International Obesity Task Force (IOTF) [18]. The conversion of BMI to z-score BMI was performed base on the WHO Growth Reference using WHO AnthroPlus (https://www.who.int/growthref/tools/en/). Waist circumference was measured using standardised procedures by placing tape between midway of the hip bone and the bottom of ribs and wrapping it around the child’s waist. Waist circumference for adolescent girl ≥ 80 cm and for boy ≥ 90 cm indicated abdominal obesity [19].

As previously described [3], blood pressure was reported by averaging three measurements after participants have been resting for 10 minutes in a sitting down position. We regularly calibrated the sphygmomanometers and used appropriate cuffs. The Clinical Practice Guidelines for Screening and Management of High Blood Pressure in Children and Adolescents by The American Academy of Pediatrics 2017 has been used as a guideline to define elevated blood pressure, including hypertension. Elevated blood pressure is defined when systolic blood pressure is ≥120 mmHg, irrespective of diastolic blood pressure. This applies for adolescents aged ≥13 years [20].

A total of 10 ml blood was collected after overnight fasting to measure serum concentrations of triglyceride, low-density lipoprotein (LDL) cholesterol, high-density lipoprotein (HDL) cholesterol, fasting blood glucose, insulin, and hemoglobin A1c (HBA1c). Fasting plasma lipid profile was measured using enzymatic assays. Increased risk of diabetes and insulin resistance was assessed using HBA1c, fasting plasma glucose, fasting insulin and homeostasis model assessment of insulin resistance (HOMA-IR). Fasting plasma insulin was measured using immunoassay, while the fasting plasma glucose will be measured using the hexokinase method. HOMA-IR was calculated from fasting plasma glucose and insulin obtained [21].

Trained research assistants measured dietary intake using a semi-quantitative food frequency questionnaire (SQ-FFQ) that has been previously used in obese adolescents in Indonesia [22]. Dietary questionnaires were analysed using NutriSurvey for Indonesian food database (EBISpro). We calculated a daily requirement intake among obese adolescents based on the recommended dietary allowance of Ministry of Health, Indonesia [23]. But dietary recommendations for children and adolescents from the American Heart Association (AHA) were established as a guide for both primordial and primary prevention of cardiovascular disease beginning at a young age [24]. For 14 to 18 years, the dietary recommendations differ between male and female adolescents. Further, we used a WHO guideline for maximum sugar intake (Table 1) [25].

**Table 1:** Daily estimated calories and recommendations for adolescents aged 14 to 18 years

	Female	Male
Calories, kcal	1800	2200
Fat, % kcal	25–35% kcal	
Fibre, g	29	38
Sodium, mg	< 2300	
Potassium, mg	< 4700	
Sugar, g^a^	50	

^a^WHO recommends a maximum to under 10% of total calories to reduce risk of unhealthy weight gain and dental caries, which equals to a maximum consumption of 50 g of sugar per day assuming a calorie intake of 2000 kcal

### Statistical analysis

Baseline data was described using mean and standard deviation or median and interquartile range or proportions as appropriate. Multivariable linear regression analysis was performed to determine the correlation between dietary intake (fibre, sugar and fat intakes) and the cardiometabolic risk factors (impaired lipid profile and glycemic control) and to adjust for potential confounders. Age, body mass index, gender, smoking status, physical activity and blood pressure were selected a priori as potential confounders. We conducted several multivariable linear regression models for each exposure-outcome association. Model 1 is the unadjusted correlation. In model 2, associations were adjusted for age and body mass index. Model 3 included adjustments for confounders as in model 2, and additional adjustments for gender. Model 4 included adjustments for confounders as in model 3, and additional adjustments for smoking status, physical activity score, and blood pressure. Data analysis was performed using IBM SPSS Statistics version 25. A *p* value < 0.05 is considered statistically significant.

## Results

### Study population and demographics

A total of 4268 students in seven public and three private high schools in Yogyakarta were screened, 298 (7%) of whom were classified as obese based on WHO, IOTF and CDC criteria. Blood samples were taken from 229 (76.8%) of those classified as obese. Therefore, we recruited 229 obese adolescents at public and private high schools in Yogyakarta, Indonesia. Demographic characteristics were described in Table 2. Only 179 adolescents voluntarily completed a semi-quantitative food frequency questionnaire (SQ-FFQ), and therefore were included in analyses of dietary intake (Table 2).

**Table 2:** Descriptive characteristics of obese adolescents aged 15–18 years in Yogyakarta-Indonesia (*n* = 179)

	Total (*n* = 179)			Males (*n* = 101)			Females (*n* = 78)		
	Median	25th Percentiles	75th Percentiles	Median	25th Percentiles	75th Percentiles	Median	25th Percentiles	75th Percentiles
Age	16.4	15.9	16.9	16.3	15.8	16.8	16.5	16	17
Height (cm)	163.3	155.5	169.3	167.5	164	173.1	155.1	152.4	159.4
Weight (kg)	84.6	77.4	95.7	91.5	83.6	101	78.1	72.6	81.9
BMI (z-score)	2.5	2.3	2.9	2.6	2.3	3	2.4	2.3	2.7
Waist to height ratio	0.56	0.53	0.60	0.58	0.54	0.61	0.55	0.52	0.58
LDL Cholesterol Direct (mg/dL)	117	97.7	142	120	103	142	113	95	140
HDL Cholesterol (mg/dL)	44	39	51	41	37	48	49	40.5	53.5
Cholesterol (mg/dL)	174	155.7	197	175	157	196.5	171	153.5	199.5
Triglyceride (mg/dL)	114	87.2	162.5	127	95	174.5	100	76.5	138.5
Fasting plasma glucose (mg/dL)	85	81	90	86	82	90	85	79.7	89.2
HbA1c (%)	5.2	5	5.3	5.2	5.1	5.4	5.1	4.9	5.3
HOMA-IR	7.1	4.7	10.6	7.4	4.9	12.2	5.9	4.1	8.9
Insulin μIU/ml	31.8	22.1	49.2	36.5	24.1	56.2	29.9	19.2	44.8
Systolic blood pressure	115	107	123	119	110	126	111.5	103.7	119
Diastolic blood pressure	73	69	80	72	68	80	74	70	80
IPAQ	546.5	198	1350	677.6	246	1836.7	395.5	104.1	1059.7

*BMI* Body mass index, *LDL* Low density lipoprotein, *HDL* High density lipoprotein, *HbA1c* Glycated hemoglobin A1c, *HOMA-IR* Homeostatic model assessment of insulin resistance, *IPAQ* International physical activity questionnaire

### Dietary intake

**Fig. 1:**
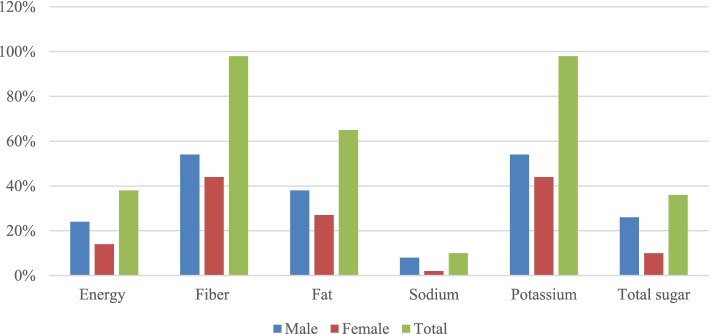
Proportion (%) of obese adolescents’ who do not meet recommendations for energy and nutrient intakes relevant to cardiometabolic health (*n* = 179). Dietary recommendations for children and adolescents from the American Heart Association (AHA) were established as a guide for both primordial and primary prevention of cardiovascular disease beginning at a young age (Gidding et al., AHA 2005). WHO Guideline: Sugars intake for adults and children (WHO March 2015). Energy (kJ) and (g) for other nutrients

Table 3 illustrates daily energy requirement and percentage of energy to recommended dietary allowance among obese adolescents in Indonesia. Compared to dietary recommendations for children and adolescents from the American Heart Association and WHO guideline, 98% of participants had inadequate intake of fibre and 65% had excess intake of fat and excess sugar intake (36%) (Fig. 1).

**Table 3:** Dietary intake among obese adolescents in Yogyakarta, Indonesia (*n* = 179)

	Total (*n* = 179)			Males (*n* = 101)			Females (*n* = 78)		
	Median	25th Percentiles	75th Percentiles	Median	25th Percentiles	75th Percentiles	Median	25th Percentiles	75th Percentiles
Energy (kcal)	1864	1305	2375	2011	1677	2641	1431	1149	1963
Protein (g)	52.5	39.1	73	60.3	47.5	78.1	42.6	32	55.4
Fat (g)	49.5	35.2	69.7	55.0	37.2	76.1	43.7	32.5	59.4
Saturated Fat (g)	13.1	7	23.2	12.4	7	24.6	13.1	6.9	21.2
PUFA (g)	3.5	2.2	5.4	4.0	2.7	6.5	2.6	2	4.2
Carbohydrate (g)	299.2	197	390.6	339.5	271.9	438.5	212.8	150.2	312.6
Total Sugar (g)	33.0	14.3	66.6	45.8	20.5	77.1	26.3	11.6	46.5
Dietary Fibre (g)	9.2	5.9	14.3	10.5	7.2	16.5	7.2	4.4	11.9
Sodium (mg)	750	477	1183	830	560	1367	698	380	972
Potassium (mg)	1336	747	1984	1503	911	2139	1015	606	1712
% CHO to RDA^a^	80	58	108	85	68	109	71	50	104
% Fibre to RDA^a^	27	18	42	28	20	44	25	15	41
% Protein to RDA^a^	74	54	98	80	63	104	65	49	85
% Total fat to RDA^a^	64	45	87	65	43	69	62	46	84
% Energy from Protein	12	10	14	12	10	13	12	10	14
% Energy from CHO	63	56	69	65	60	71	60	53	67
% of CHO as Sugar	13	7	21	14	7	21	13	5	19
% Energy from Fat	25	20	31	24	17	29	27	21	55

*PUFA* Poly-unsaturated fatty acid, *CHO* Carbohydrate

^a^The recommended dietary allowance (RDA) is based on Indonesia’s Ministry of Health Recommendations. Number 28. 2019

### Relationship between dietary intake and CVD risk factors

There was a statistically significant correlation between fibre intake and level of HDL cholesterol (β = 0.165; *p* = 0.033) in the multivariable linear regression analysis (Table 4). Further, we might found a correlation between sugar intake with HBA1c concentrations, but this was not statistically significant (β = 0.173; *p* = 0.055) (Table 5).

**Table 4:** Multivariable linear regression of association between sugar, fat and fibre intake with HDL cholesterol and triglyceride

	Model 1		Model 2		Model 3		Model 4	
	β	*p* value	β	*p* value	β	*p* value	β	*p* value
**Sugar intake**								
HDL cholesterol	−0.039	0.648	−0.031	0.718	−0.008	0.926	0.37	0.441
Triglyceride	0.057	0.50	0.046	0.541	0.030	0.726	0.018	0.834
**Fat intake**								
HDL cholesterol	0.059	0.491	0.059	0.493	0.087	0.298	0.070	0.391
Triglyceride	−0.094	0.276	−0.093	0.276	−0.113	0.182	−0.107	0.196
**Fibre intake**								
HDL cholesterol	0.215	0.008	0.207	0.01	0.175	0.027	**0.165**	**0.033**
Triglyceride	0.186	0.022	0.175	0.029	0.152	0.057	0.151	0.053

Model 1 is the unadjusted correlation. In model 2, associations were adjusted for age and body mass index. Model 3 included adjustments for confounders as in model 2, and additional adjustments for potential mediation by gender. Model 4 included adjustments for confounders as in model 3, and additional adjustments for potential mediation by smoking status, physical activity score, and blood pressure

*β* Standardised beta

**Table 5:** Multivariable linear regression of association between sugar, fat and fibre intake with levels of fasting plasma glucose and HBA1c

	Model 1		Model 2		Model 3		Model 4	
	β	*p* value	β	*p* value	β	*p* value	β	*p* value
**Sugar intake**								
HbA1c	0.164	0.054	0.158	0.064	0.161	0.061	0.173	0.055
Fasting plasma glucose	0.104	0.223	0.105	0.222	0111	0.198	0.138	0.124
**Fat intake**								
HbA1c	0.108	0.314	0.086	0.312	0.090	0.295	0.089	0.309
Fasting plasma glucose	0.099	0.253	0.098	0.255	0.106	0.223	0.106	0.224
**Fibre intake**								
HbA1c	0.013	0.872	0.007	0.085	0.011	0.891	0.014	0.866
Fasting plasma glucose	0.004	0.957	0.005	0.950	0.014	0.867	0.016	0.842

Model 1 is the unadjusted correlation. In model 2, associations were adjusted for age and body mass index. Model 3 included adjustments for confounders as in model 2, and additional adjustments for potential mediation by gender. Model 4 included adjustments for confounders as in model 3, and additional adjustments for potential mediation by smoking status, physical activity score, and blood pressure

*β* Standardised beta

## Discussion

This study explored the relationship of dietary intake and cardiovascular disease risks in 156 obese adolescents in Indonesia. This study demonstrates a high proportion of obese Indonesian adolescents with unhealthy diet. Fibre intake was correlated with the level of HDL cholesterol. Further, sugar intake might be correlated with risk of diabetes.

Obesity and cardiovascular disease are urgent public health priorities. Only a third of genetic influences play a role on the development of obesity. Majority of risk factors for developing obesity are modifiable; such as eating habits and sedentary behavior [26]. These modifiable risk factors can track from adolescence in adulthood and lead to cardiovascular disease in older age.

Suboptimal dietary intake comprises of high total fat, high total sugar, and low fibre intake. This present study demonstrated that unhealthy dietary intake among obese adolescents in Indonesia was prevalent demonstrating most of those consumed very low fibre, high total sugar, and high fat particularly saturated fatty acid. This dietary intake might lead to obesity and other obesity related cardiovascular diseases. Indeed, this study shows that low fibre intake significantly correlated with low HDL cholesterol in obese adolescents after adjusting for other potential confounding factors. While sugar intake might be positively correlated with levels of fasting glucose. High sugar and fat intakes may cause increased production of lipids, secretion of very low-density lipoproteins, accumulated fatty acids, and reduced oxidation that lead to atherosclerosis [27].

Fat is an essential source of energy, fatty acid, and fat-soluble vitamins. However, fat intake might cause dyslipidemia that led to the development of atherosclerosis and myocardial infarction [9]. Saturated fatty acid may result in increased cardiovascular disease, while polyunsaturated fatty acid contributes to decreased serum cholesterol and decreased incidence of coronary-artery disease [28]. Results of previous studies reveal that high fat intake contributing to increased LDL cholesterol and reduced HDL cholesterol [9]. In this study, we could not prove that fat intake correlated with cardiovascular disease risks. This might because of small sample size, so that the contradictory results are common.

High sugar intake is considered to be associated with diabetes, cardiovascular disease, obesity, and high blood pressure [9, 29]. A previous study on dietary intake in Korean adults showed that consumption of high fat, sugar and low fibre associated with incidence of obesity [30]. High sugar intake positively correlated with risk of diabetes in this present study. This has a general agreement with previous study that high sugar intake associated with diabetes, obesity, and other cardiovascular risks [11, 31]. This present study also showed that high sugar intake did not correlated with the occurrence of insulin resistance. Our study corresponds to a study conducted among students aged 10 to 17 years in a developed country showing that intake of sugar and fibre was not associated with reduced in cardio-metabolic risk factors including hypertension and insulin resistance [31]. Further, in adult Chinese population, current non-smoking status, a diet rich in vegetables and fruits and high physical activity were independently associated with reduced risks of major coronary events and ischemic stroke [32]. Therefore, in this study we also made an effort to adjust for other factors that might be associated with the development of cardiovascular risks including smoking status and physical activities.

An unhealthy diet intake is strongly associated with cardiovascular diseases. Most obese adolescents in this study had low fibre intake (98%). This is comparable to the results of the Riskesdas surveys revealing that the majority of Indonesians (94%) did not consume an adequate amount of fruits and vegetables, which is five portions on seven continuous days [23].

Dietary recommendations for children and adolescents included an adequate amount of fibre, sugar, fat, other macro and micronutrients. These recommendations aim at achieving adequate nutrition for optimal growth and development. Malnutrition with an imbalanced intake of nutrients in terms of quality and quantity including macro and micronutrient can negatively affect child development and increase cardiovascular risk in later life [33]. Evidence on the association of dietary intake and cardiovascular disease risk among children are limited. Therefore, this present study might shine a light on enriching the researches on the correlation between dietary intake and cardiovascular disease risks in obese adolescents. Evidence arises from this study could contribute to the development of an effective strategy for preventing cardiovascular disease risks later in life.

There are some limitations of this study. First, this is cross-sectional study, so relationships described between exposure and outcome are not causal. There is also the possibility dietary intakes were underestimated by the FFQ methods. It is well documented that the methods used for dietary assessment are prone to recall bias. The responses of the obese adolescents may also be impacted by social desirability bias. Second, since this study was only performed in a city of Yogyakarta, the results of this study may not be generalizable to other obese adolescents in Indonesia. Third, since the sample size of this study was quite small, we might found insignificant relationship between diet and CVD risk factors.

This study shows that intake of unhealthy nutrients among obese adolescents in Yogyakarta, Indonesia might be prevalent. There was relationship observed between intake of nutrients of concern and cardiometabolic risk factors among this sample of obese adolescents. This study potentially provides a model for the correlation of dietary intake and obese-related disease in adolescents in a low- and middle-income country and broader strategies to prevent cardiovascular disease in adulthood. This study provides rationale for policy makers in Indonesia to consider obesity prevention and health promotion policies and programs targeting children and adolescents to prevent non-communicable disease burden in future.

## Supplementary Information

**Additional file 1.**

**Additional file 2: Supplementary table 1.** The criteria of obese based on WHO, CDC, and IOTF references.

## Data Availability

All data generated or analyzed during this study are included in this published article [and its supplementary information files].

## Abbreviations

BMI: Body mass index
HDL: High-density lipoprotein cholesterol
LDL: Low-density lipoprotein cholesterol
HbA1c: Glycated hemoglobin A1c
WHO: World health organization
CDC: the Centers for disease control and prevention
IOTF: the International obesity task force
HOMA-IR: Homeostasis model assessment of insulin resistance
SQ-FFQ: A semi-quantitative food frequency questionnaire
AHA: American heart association
RDA: Recommended dietary allowance
PUFA: Poly-unsaturated fatty acid
CHO: Carbohydrate
