# Supplementary material for: The association between dietary intake and cardiometabolic risk factors among obese adolescents in Indonesia
Source: BMC Pediatr. 2022 May 12;22:273. doi: 10.1186/s12887-022-03341-y (PMC9097319; doi:10.1186/s12887-022-03341-y)
Supplement: Supplementary file 2 — Additional file 2: Supplementary table 1. The criteria of obese based on WHO, CDC, and IOTF references. [file 12887_2022_3341_MOESM2_ESM.docx]

Supplementary table 1. The criteria of obese based on WHO, CDC, and IOTF references

| WHO | CDC | IOTF |
| --- | --- | --- |
| The WHO growth charts for children aged 5 to 19 years in 2007, and based on this growth standard overweight and obesity were estimated. Obesity if BMI>+2SD, overweight if BMI>+ 1 standard deviation (SD), normal weight between +1SD and -1SD, thinness if <-2SD, and severe thinness if BMI<-3SD | The percentile ranking of BMI relative to CDC-BMI for-age-growth charts (girls/boys from 2 to 19 years) and calculated underweight (less of 5th percentiles), overweight (excess of 85th percentiles to 95th percentiles), and obesity (excess of 95th percentiles) | BMI values for ages 2 to 18 years BMI cut-off points of 30 kg/m^2^ were considered for obese in adults as well as in children and adolescents |

WHO=World Health Organization; CDC= Centers for Disease Control and Prevention; IOFT= the International Obesity Task Force; BMI= body mass index; SD=standard deviation.
